# Arthritogenic *Alphavirus* Capsid Protein

**DOI:** 10.3390/life11030230

**Published:** 2021-03-11

**Authors:** Shambhavi Rao, Adam Taylor

**Affiliations:** Emerging Viruses, Inflammation and Therapeutics Research Group, Gold Coast Campus, Menzies Health Institute Queensland Griffith University, Southport, QLD 4222, Australia; shambhavi.rao@griffithuni.edu.au

**Keywords:** capsid, old world alphaviruses, antivirals, vaccines

## Abstract

In the past two decades Old World and arthritogenic alphavirus have been responsible for epidemics of polyarthritis, causing high morbidity and becoming a major public health concern. The multifunctional arthritogenic alphavirus capsid protein is crucial for viral infection. Capsid protein has roles in genome encapsulation, budding and virion assembly. Its role in multiple infection processes makes capsid protein an attractive target to exploit in combating alphaviral infection. In this review, we summarize the function of arthritogenic alphavirus capsid protein, and describe studies that have used capsid protein to develop novel arthritogenic alphavirus therapeutic and diagnostic strategies.

## 1. Overview

Alphaviruses are positive single stranded RNA viruses, belonging to virus family *Togaviridae* [1]. They are vector borne viruses circulated by mosquitoes, typically of the genus *Aedes,* and are distributed globally [2]. Alphaviruses are split into two sub-groups: The New World (NW) alphaviruses and the Old World (OW) alphaviruses [3]. This classification is based on the historical geographic distribution of these viruses and the clinical outcomes of disease. NW alphaviruses typically cause disease with neurological implications and are thus referred to as encephalitic alphaviruses. OW alphaviruses typically cause rheumatic disease and are thus referred to as arthritogenic alphaviruses [4,5].

NW alphaviruses have evolved separately from the OW alphaviruses [1] and have not only modified their envelope glycoproteins and replicative machinery for replication in particular species of hosts and vectors but have also evolved different mechanisms of modifying the cellular environment and interfering with the development of an antiviral response [6,7]. Recently, probing techniques identified structural elements within OW and NW alphaviral genomes suggesting that these RNAs are highly divergent structurally despite similar genomic architecture and sequence conservation [8].

The OW category comprises polyarthritic alphaviruses endemic to Asia, Europe, Australia, and Africa [9]. Arthritogenic alphaviruses are now distributed globally [1]. Ross River virus (RRV) and Barmah Forest virus (BFV) are endemic to Australia and Islands of the Pacific [10,11], O’nyong’nyong virus (ONNV) is found in Africa [12] and Mayaro virus (MAYV) in South and Central America [13]. In the past two decades, OW alphaviruses such as Chikungunya virus (CHIKV), Sindbis virus (SINV), RRV, MAYV, BFV, and ONNV have been responsible for epidemics of public health concern and have caused high morbidity characterized by polyarthritis. Symptoms can progress to debilitating arthritis lasting months or years causing chronic pain [14]. CHIKV alone has emerged as a human pathogen of global concern over the past decade. CHIKV outbreaks have occurred in Southeast Asia, the Indian Ocean islands of Mauritius, La Réunion (270,000 cases in 2005–2006) and the Seychelles and India (3–4 million estimated cases 2005–2011) [15,16,17]. In 2013 CHIKV also spread to the Americas causing over 2 million cases of infection through the end of 2017 [18]. The major reasons behind the epidemic potential of CHIKV and other arthritogenic alphaviruses are the increasing range of *Aedes* mosquitoes, urbanization, vector adaptation, climate change and cross-country travel, making these viruses an issue of global health concern [19].

Alphavirus capsid protein is a versatile protein that actively participates in genome encapsulation, virus budding and virion assembly [1,20,21]. Capsid protein is a multifunctional protein. Capsid protein comprises two domains: the N-terminal domain and C-terminal domain. The N-terminal domain is highly disordered and contains a stretch of positively charged amino acid residues [22]. The N-terminus aids in the binding of capsid protein with viral genomic RNA, thus allowing RNA encapsidation and formation of nucleocapsid protein cores [23]. Amino acid residues in the N-terminal of capsid proteins are involved in capsomere interactions [24,25]. The C-terminal domain has a chymotrypsin-like serine protease fold containing a conserved hydrophobic pocket that is involved in interaction with the cytoplasmic domain of envelope protein 2 (E2) [21,26,27,28,29]. The C-terminal has autoproteolytic activity and cleaves itself from the N-terminus of the structural polyprotein, playing a critical role in processing of the structural polyprotein and the viral life cycle [30]. The multifunctional capsid protein is crucial for viral replication and the potential to inhibit its functions has attracted attention as a target for therapeutic treatment.

This review investigates the recent advancements in arthritogenic alphaviral capsid protein research. We examine the multifunctional nature of arthritogenic alphaviral capsid proteins and how this has aided the design and development of new strategies to combat debilitating alphaviral infections.

## 2. Alphaviral Virion and Genome Structure

Alphaviruses are single stranded positive-sense RNA viruses [1]. They are enveloped viruses that measure ~700 Å in diameter [24,31]. The alphaviral RNA genome is ~12 kb in length [32]. The coding sequence consists of two open reading frames (ORFs) where the N-terminal ORF encodes the non-structural polyprotein and the C-terminal ORF encodes the structural polyprotein [1,33]. The first ORF which is about two-thirds of the genome codes for the non-structural polyprotein. Non-structural proteins are involved in enzymatic functions necessary for viral replication, antagonize the host immune system, and modulate cellular transcription and translation [34]. The second ORF encodes the structural proteins. The ORF is translated as a polyprotein and is co/post-translationally cleaved into the individual structural proteins by host and viral proteases. Specifically, the capsid protein, found at the N-terminus of the structural polyprotein, is an autoprotease and cleaves itself from the growing polypeptide [35]. Cryo-electron microscopy and crystallographic studies on alphaviruses and their proteins has revealed important detail about the molecular organization of the virion [26,36,37]. Alphaviruses are composed of a host derived lipid bilayer associated with two glycoproteins, a nucleocapsid containing capsid proteins and a 49S RNA molecule [1]. The outer envelope of the virus contains 80 envelope protein 1 and envelop protein 2 (E1, E2) heterodimers [1,38,39,40].

The alphaviral nucleocapsid is 400 Å in diameter and is made up of 240 copies of the capsid protein organized in a T = 4 icosahedral arrangement [41,42]. The envelope proteins and the nucleocapsid are believed to interact through C-terminal residues of E2 that are exposed on the inner surface of the lipid bilayer and the capsid protein C-terminal domain [26,43]. The E1 glycoprotein is involved in the formation of the icosahedral shell of the virus particle and membrane fusion during virus entry into the host cell [44]. Molecular interplay between E2 and the hydrophobic pocket present in the C-terminal domain of capsid protein plays an essential role in the alphavirus budding process [26,45]. X-ray crystallographic and cryo-electron microscopy (cryo-EM) studies have provided detail of the interactions of capsid with E1 and E2 proteins [26,43]. Crystallization of recombinant SINV capsid protein identified amino acids 108 to 111 bind a specific hydrophobic pocket in neighbouring molecules [26]. Binding of capsid residues 108 and 110 into the alphaviral core protein hydrophobic pocket is suggested to mimic the binding of E2 protein’s C-terminal residues in the hydrophobic pocket. Mutational analysis of capsid residues 108 and 110 support their role in alphaviral nucleocapsid assembly [26].

## 3. Alphaviral Capsid Protein

The alphavirus genome is a single capped polyadenylated RNA, termed the 49S RNA [1]. The 49S RNA serves as mRNA, becoming translated into the non-structural polyprotein. 49S RNA also serves as a template for transcription of the 26S sub-genomic mRNA, encoding the structural polyprotein referred to as p130 [46]. The sub-genomic 26S RNA is capped and polyadenylated [32,47]. The structural proteins are required for virion entry, nucleocapsid assembly and virus budding from the host cell. The capsid protein is located at the N-terminus of p130. The capsid protein of the prototype species of alphavirus, Sindbis virus (SINV), consists of 263 amino acids [47]. Once the p130 polyprotein is translated, the capsid protein is auto-proteolytically cleaved and released into the host cytoplasm [48].

The C-terminal domain of alphaviral capsid protein serves as a chymotrypsin-like serine protease, which is responsible for a single cleavage event to separate the capsid protein from the rest of the structural protein [30,49,50,51]. Autoproteolytic cleavage occurs between tryptophan 267 and serine 268 of the polyprotein and releases the capsid protein [52]. The cleavage exposes a signal sequence at the N-terminus that allows the remainder of the structural polyprotein (p130) to be inserted into the endoplasmic reticulum (ER), there undergoing processing of other structural components of the alphaviral envelope [33,48,53]. The C-terminal domain of capsid protein is responsible for formation of the pentameric and hexameric capsomeres of the nucleocapsid [33,54]. The C-terminal domain is highly conserved in alphaviruses [1].

The N terminus of SINV capsid has two major regions: region I (amino acid residues range: 1–80) and region II (amino acid residues range: 81–113), which are involved in encapsulation of the genomic RNA [55]. Region I is postulated to be involved in the dimerization of capsid proteins through coiled coil interactions [25]. Region II is primarily involved in interaction with RNA packaging signals [20,56]. The N-terminal domain is less conserved and intrinsically disordered with a high degree of positive charge [20]. Despite this there are regions of high conservation within the N-terminal responsible for binding the viral RNA genome, dimerization, nuclear import and export of capsid protein and in NW alphaviruses shutting off host transcription [20,57].

Structural studies have revealed that the basic molecular architecture of the active site and catalytic site of serine proteases are highly conserved, including that within alphavirus capsid protein [30,58]. Recent studies on encephalitic alphaviruses have elucidated that the capsid protein N-terminal comprises four separate subdomains, unlike the N-terminal of arthritogenic alphaviruses [57]. The subdomains have regulatory functions in particle assembly, the specificity of RNA encapsidation, nucleocapsid stability and RNA presentation for encapsidation. Encephalitic alphaviruses like Venezuelan equine encephalitis virus (VEEV), Western equine encephalitis virus (WEEV), etc. use the capsid protein to shut off host transcription [7,59].

The crystal structure of arthritogenic alphavirus CHIKV capsid protein has been determined at 2.2 Å [37]. This structure reveals the chymotrypsin-like protease fold with a conserved hydrophobic pocket in the C-terminal of capsid protein for interaction with the cytoplasmic tail of E2 [33,34,35,36,37]. This interaction is essential for virus budding [27]. A study by Sharma et al. used molecular docking, surface plasmon resonance (SPR) and fluorescence spectroscopy to further characterize capsid protein interactions involving the hydrophobic pocket [27]. This study determined that mandelic acid and ethyl 3 aminobenzoate bind to the conserved hydrophobic pocket in capsid protein and could serve as the basis for antiviral development [27]. Mandelic acid and ethyl 3 aminobenzoate were selected based on their structural similarity to picolinic acid (PCA), which was shown previously to target the hydrophobic pocket of capsid protein [60]. These interactions are predicted to disrupt the E2-capsid protein molecular interactions, and consequently inhibit virus budding and replication [60].

Alphaviral capsid protein is ~29 kDa and contains both a nuclear localization signal (NLS) and nuclear export signal (NES) allowing active transport to and from the nucleus [61,62,63]. This is despite capsid protein being small enough for passive transport through nuclear pores. The CHIKV capsid protein NLS has been mapped to amino acids 60–99 and NES mapped to residues 143–155 [63]. Bidirectional transport of molecules between the nucleus and the cytoplasm occurs through the nuclear pore complex, a supra-molecular structure of the nuclear envelope [64]. The active nuclear import of cytoplasmic proteins is mediated by karyopherins and their export by exportins [65]. A 2013 study demonstrated that CHIKV capsid protein binds to karyopherin α (Karα) during nuclear translocation, and that the Karα4 C-terminal NLS binding domain is sufficient for this interaction [63].

A study investigating the NES in the N-terminal region of CHIKV capsid protein identified chromosomal maintenance 1 (CRM1) export adaptor protein as a mediator of capsid protein export from the nucleus [63]. This study also reported that the NLS was located within the N-terminal region of CHIKV capsid protein. This study further elucidated that the capsid protein did not inhibit host nuclear import, unlike NW alphaviral capsid protein. However, mutations in the NES of CHIKV capsid protein blocked host protein movement to the nucleus [66]. Capsid protein is also able to translocate to the host cell nucleolus [67,68]. In NW encephalitic alphaviruses, nuclear translocation induces host cell transcriptional shutoff; however, the role of capsid protein nucleolar trafficking in arthritogenic alphaviruses remains unclear. Further investigation is required to fully understand the role of capsid protein nuclear trafficking in arthritogenic alphaviral replication.

## 4. Arthritogenic Alphaviral Capsid Protein in Vaccine Design

As the antigenic diversity of individual arthritogenic alphaviruses is limited and the viruses are incapable of re-infection, a vaccine would be an effective solution to counteract disease and prevent circulation amongst populations [69]. Several vaccine strategies including inactivated vaccines [70,71,72], live attenuated vaccines [67,73], alphavirus chimeras [74], recombinant vaccines [75], DNA vaccines [76], subunit vaccines [77] and more recently virus like particles (VLPs) [78] have been employed to develop an effective alphavirus vaccine, yet there is no licensed vaccine currently available.

A recent study used recombinant EGFP-tagged capsid protein and CHIKV infectious clones, to identify the nucleolar localization sequence (NoLS) of capsid protein in the N-terminal region [67]. Site directed mutagenesis of the NoLS reduced the efficiency of capsid protein nuclear import. Importantly for vaccine development, mice inoculated with CHIKV containing the mutated NoLS developed no CHIKV disease. Mice inoculated with the NoLS mutant virus were protected from disease when challenged with CHIKV and showed reduced viremia when challenged with related arthritogenic alphaviruses, indicating a cross-protective effect [67]. A follow up study in 2020 used the capsid NoLS mutant live-attenuated CHIKV vaccine candidate to test more stable and efficient ways of delivery of the vaccine candidate [79].

A study in 2019 reported the generation of a live-attenuated CHIKV vaccine by deleting the entire capsid protein. The mutant when grown in BHK cell lines had antigenic properties like those of wild type CHIKV. Vaccination of both immunocompetent and immunocompromised mice resulted in complete protection against CHIKV challenge [80].

Capsid protein is critical to the generation of a recombinant live-attenuated measles vaccine expressing CHIKV VLPs [81]. The VLPs comprise CHIKV envelop and capsid proteins. Immunization of mice susceptible to measles virus produced high titres of CHIKV neutralizing antibodies accompanied by viral specific cellular responses. A single immunization with this vaccine candidate protected all mice from lethal CHIKV challenge, and passive transfer of immune sera conferred protection to naïve mice [81].

An investigation into viral protein sequences involved in tissue tropism of viral proteins identified the presence of a conserved sequence, VAIVLGG, in alphaviruses [82]. This amino acid sequence was located in capsid protein. The sequence is not found in the human proteome, which raises the possibility of using the sequence to develop an antigen for producing cross protective vaccines, effective against a wide range of alphaviruses [82]. Serum from CHIKV-infected macaques has been used to demonstrate the importance of capsid and virus structural proteins in the protective immune response [83]. Sera collected at different time-points post infection demonstrated a long-lived capsid protein antibody response and epitopes within capsid protein important for antibody recognition. Results of this study demonstrate the importance of the capsid protein in the adaptive immune response to CHIKV infection and will aid vaccine design [83].

One study used baculovirus vectors to produce high levels of alphavirus core-like particles (CLPs) in insect cells by expression of CHIKV and aquatic salmonid alphavirus (SAV) capsid proteins [84]. The CLPs were reported to localize in dense nuclear bodies within the infected cell nucleus and were purified easily through cell lysis, sonication and low-speed centrifugation. This study also reported the successful tagging of an immunogenic epitope from the alphavirus E2 glycoprotein to the N-terminus of the capsid protein without disrupting CLP self-assembly. Immunogenic epitope-tagged alphavirus CLPs produced in insect cells is a simple and more stable vaccine development alternative [84].

## 5. Arthritogenic Alphaviral Capsid as a Therapeutic Target

No licensed drug exists for the treatment of arthritogenic alphaviral infection. Current treatments such as administration of non-steroidal anti-inflammatory drugs (NSAIDS), only alleviates the symptoms of disease. There is an urgent need to develop inhibitors that act on virus-specific targets. It is therefore important to understand the underlying mechanisms of viral replication to aid antiviral development. Studies to uncover relevant drug targets to treat infection are ongoing. Aggarwal et al. developed a fluorescence resonance energy transfer (FRET)-based proteolytic assay for high throughput screening (HTS) of capsid protease inhibitors [85]. A FRET peptide substrate was derived from the cleavage site in the CHIKV structural polyprotein [85]. The study screened chemical libraries and compounds including tryptophan, tryptamine compounds, different serine protease inhibitors like phenylmethanesulfonyl fluoride (PMSF), benzamidine, N-p-Tosyl-L-phenylalanine chloromethyl ketone (TPCK) and 3-acetylindole but, failed to recognize compounds that inhibited CHIKV capsid protease activity. HTS technologies can be highly efficient in recognizing key players in viral replication.

Capsid protease remains a promising target for antiviral drug discovery. A 2020 study reported antiviral molecules targeting arthritogenic alphavirus capsid protein proteolytic activity [86]. Structure assisted drug repositioning was used to identify three molecules P1, P4-Di(adenosine-5′) tetraphosphate (AP4), Eptifibatide acetate (EAC) and Paromomycin sulphate (PSU) as potential capsid protease inhibitors. FRET was used to confirm the antiviral activity of these molecules. These inhibitors had no effects on viral RNA synthesis and treatment of cells with inhibitors reduced levels of capsid protein in infected cells. Each inhibitor significantly reduced the production of infectious virus, particularly during the later stage of the viral life cycle when capsid protein production is at its maximum. Furthermore, atomic structure determination of CHIKV capsid protein in complex with the inhibitors is in progress, allowing rational drug design to increase antiviral potency of the identified molecules [86].

A study in 2015 characterized an arthritogenic alphaviral capsid protein insertion mutation which affected the nucleocapsid stability of the virus. An amino acid insertion after position 186 in capsid produced a temperature sensitive phenotype. This insertion was located at the interface of capsid proteins assembled in nucleocapsid, suggesting that this region was essential for stabilizing the nucleocapsid. Further studies will determine the functional significance of this finding during virus infection and the potential to exploit this interaction therapeutically [87].

A study in 2016 revealed the potential of pyridine ring containing compounds, such as PCA, as arthritogenic alphaviruses capsid protein targeting antivirals. PCA was shown by molecular docking, isothermal titration calorimetry, surface plasmon resonance and fluorescence spectroscopy to bind the hydrophobic pocket of CHIKV capsid protein and inhibit viral replication in Vero cells. The results confirmed the anti-alphavirus capacity of pyridine ring compounds and their potential use as a stage for therapeutic development [60].

A similar study was reported in 2017 using small heterocyclic molecules, such as piperazine, and exploiting their ability to bind the hydrophobic pocket of capsid protein as a potential pharmaco-therapeutic. The antiviral activity of piperazine against CHIKV was investigated by plaque reduction and immunofluorescence assays. Piperzine effectively inhibited CHIKV growth at a concentration of 6 mM with no cytotoxicity observed at this concentration. Thus, the capsid-piperzine complex may serve as a lead scaffold for structure-based design of piperazine derivative alphaviral inhibitors [88].

Formation of the alphaviral nucleocapsid cores, containing viral nucleic acid and the capsid protein, is an essential molecular process of infection, yet the exact interactions between the two partners are not fully understood. In a 2017 study, CLIP-seq (Cross-linking immunoprecipitation sequence) was used to screen for sites of interaction between the capsid protein and genomic RNA of SINV, a model alphavirus [89]. SINV capsid protein was shown to bind specific viral RNA sequences in the cytoplasm of infected cells, but its interaction with genomic RNA in extracellular viral particles is largely non-specific. Mutation of the capsid protein-RNA interaction site inhibited viral growth. It was proposed that mutational analysis reduced stability of the incoming viral genomic RNA, suggesting a role for capsid protein in the stabilization of viral RNAs. This functional role of capsid protein during early alphaviral replication, to promote infectivity, is a novel virulence determinant that may serve as a target for drug design [89].

Berberine chloride (BBC) is a pan-alphavirus inhibitor that was identified in a replicon-based small-molecule screen [90]. BBC was shown to have antiviral capacity affecting later processes of the viral life cycle and the nucleocapsid core. Infected cells treated with BBC late in infection were unable to form stable cytoplasmic nucleocapsid cores or assembly intermediates, as assayed by gradient sedimentation. In vitro studies with recombinant capsid protein revealed that BBC perturbs core-like particle formation. BBC treatment of virus particles showed a strong decrease in alphavirus infectivity. Together, this study indicated that BBC alters alphavirus capsid protein and viral RNA interactions and oligomerization, causing defects in nucleocapsid assembly [90].

## 6. Use of Arthritogenic Alphavirus Capsid Protein in Development of Diagnostics

The clinical symptoms of arthritogenic alphavirus infection are often similar to one another. Additionally, there is significant overlap in the symptoms of arthritogenic alphavirus infection with other arbovirus infections, such as flaviviruses [91,92,93]. Flaviviruses and alphaviruses regularly share the same geographic distribution which can further hamper accurate clinical diagnosis [41]. Improved detection methods to identify arthritogenic alphaviral infection are required to improve treatment and clinical management.

CHIKV has been responsible for epidemics of debilitating arthritis [94,95,96,97,98]. Recent outbreaks (2004–2014) resulted in an estimated 1.4–6.5 million cases, with imported cases reported in nearly 40 countries [99]. The development of CHIKV specific diagnostics and research tools is highly desirable. A study from 2014 describes the development and evaluation of recombinant capsid protein based indirect IgM antibody capture micro plate enzyme linked immunosorbent assay (ELISA) for rapid and accurate diagnosis of CHIKV infection [100]. No cross-reactivity with dengue virus was observed. The findings clearly demonstrate the utility of a recombinant capsid protein based CHIKV IgM ELISA for reliable clinical diagnosis of CHIKV infection in humans [100].

A 2015 study described the generation and characterization of monoclonal antibodies (mAbs) specific to CHIKV capsid protein [101]. These antibodies were able to recognize the isolates representing the major genotypes of CHIKV, as well as several other arthritogenic alphaviruses and were reactive in a range of assays including ELISA, Western blot, immunofluorescence and immunohistochemistry (IHC) [101]. A further study describes the development of mAb-based IgM capture ELISA (MAC ELISA), which detects CHIKV-specific IgM antibodies. All of the subclones of mAbs derived from the IgG1 hybrid recognized the capsid protein of CHIKV [102].

A panel of eleven mAbs previously generated for detection of the capsid protein of CHIKV have been epitope mapped using N- and C-terminally truncated recombinant forms of CHIKV capsid protein [103]. These recombinant forms were used to recognize two putative binding regions. A smaller N-terminus truncated product of capsid protein was identified in this study which may represent an alternative translational product of the 26S sub genomic RNA. Although the functional significance of the truncated capsid protein during viral replication is unclear, anti-capsid mAbs will serve as valuable tools for further investigation of the structure and function of arthritogenic alphaviral capsid proteins [103].

Studies have successfully generated mouse anti-CHIKV mAbs targeting CHIKV E1 and capsid proteins that lack cross-reactivity towards SINV and flaviviruses like dengue and Zika virus. Two mAbs also lacked cross-reactivity with other related arthritogenic alphaviruses like ONNV, MAYV, RRV and NW alphaviruses like WEEV and VEEV. The capsid-envelope protein targeting mAbs generated by this study will be promising candidates for the development of antibody-based rapid diagnostic tests [104].

## 7. Conclusions

Arthritogenic alphaviruses are a re-emerging group of arthropod transmitted viruses that are globally widespread. Arthritogenic alphaviruses are increasing their impact on humankind with infections resulting in severe debilitating arthritis, arthralgia and myalgia. There is no licensed drug or vaccine to treat arthritogenic alphaviral infections. The alphavirus capsid is a multifunctional protein with roles in genome encapsulation, budding and virion assembly. Our increased understanding of capsid protein function throughout the infection process has led to its pivotal use in novel therapeutic strategies.

An increasing number of studies have attempted to exploit the importance of arthritogenic alphaviral capsid protein function as a target for drug and vaccine development. High throughput technologies, like (FRET)-based proteolytic assays, have helped identify lead compounds like PCA, BBC, mandelic acid and ethyl 3 aminobenzoate as potential capsid protein targeting antivirals, or scaffolds on which to further develop antiviral compounds. New vaccination strategies and biotechnological tools, such as VLPs, are being employed to produce promising vaccine candidates to prevent infection. Many capsid protein-based therapeutic strategies remain in preclinical development. Additional studies are required to confirm the effectiveness of these therapeutics. For example, given the importance of capsid protein to the protective immune response against alphaviral infection, further studies to examine the safety and immunogenicity of vaccine candidates that remove capsid protein are warranted.

Additional functions of arthritogenic alphaviral capsid protein remain undiscovered, including the role of nuclear and nucleolar host cell trafficking during infection. Detailed studies are required to fully understand the role of the multifaceted capsid protein during the infection process.

## Data Availability

Not applicable.
